# Comparative value of a simulation by gaming and a traditional teaching method to improve clinical reasoning skills necessary to detect patient deterioration: a randomized study in nursing students

**DOI:** 10.1186/s12909-020-1939-6

**Published:** 2020-02-19

**Authors:** Antonia Blanié, Michel-Ange Amorim, Dan Benhamou

**Affiliations:** 1 Centre de simulation LabForSIMS, Faculté de médecine Paris Saclay, 94275 Le Kremlin Bicêtre, France; 2 Département d’Anesthésie-Réanimation, CHU Bicêtre, 78, rue du Général Leclerc, 94275 Le Kremlin Bicêtre, France; 3 CIAMS, Univ. Paris-Sud, Université Paris-Saclay, 91405 Orsay, Cedex France; 4 CIAMS, Université d’Orléans, 45067 Orléans, France

**Keywords:** Serious games, Simulation, Clinical reasoning, Motivation, Nursing students, Deterioration, Script concordance test

## Abstract

**Background:**

Early detection and response to patient deterioration influence patient prognosis. Nursing education is therefore essential. The objective of this randomized controlled trial was to compare the respective educational value of simulation by gaming (SG) and a traditional teaching (TT) method to improve clinical reasoning (CR) skills necessary to detect patient deterioration.

**Methods:**

In a prospective multicenter study, and after consent, 2nd year nursing students were randomized into two groups:

- Simulation by gaming “SG”: the student played individually with a serious game consisting of 2 cases followed by a common debriefing with an instructor;
- Traditional Teaching “TT”: the student worked on the same cases in text paper format followed by a traditional teaching course with a PowerPoint presentation by an instructor.

CR skill was measured by script concordance tests (80 SCTs, score 0–100) immediately after the session (primary outcome) and on month later. Other outcomes included students’ satisfaction, motivation and professional impact.

**Results:**

One hundred forty-six students were randomized. Immediately after training, the SCTs scores were 59 ± 9 in SG group (*n* = 73) and 58 ± 8 in TT group (*n *= 73) (*p* = 0.43). One month later, the SCTs scores were 59 ± 10 in SG group (*n* = 65) and 58 ± 8 in TT group (*n* = 54) (*p* = 0.77). Global satisfaction and motivation were highly valued in both groups although significantly greater in the SG group (*p* < 0.05). The students declared that the training course would have a positive professional impact, with no difference between groups.

**Conclusions:**

In this study assessing nursing student CR to detect patient deterioration, no significant educational difference (SCT), neither immediate nor 1 month later, was observed between training by SG and the TT course. However, satisfaction and motivation were found to be greater with the use of SG.

**Trial registration:**

ClinicalTrials.gov; NCT03428269. Registered 30 january 2018.

## Background

Patient deterioration detection is a major healthcare problem. Indeed, acute patient clinical deterioration is often preceded by a modification of physiological parameters 6 to 24 h before the event [1, 2]*.* The association of i) early detection ii) rapidity of response and iii) quality of clinical response, influence the patient’s prognosis. Many studies have shown that delayed diagnosis of an ongoing complication increases morbidity and mortality [2, 3]. The education of nurses, who are frontline healthcare providers, is therefore essential. In French nursing institutes, theoretical training to detect patient deterioration is currently performed by traditional teaching courses and/or paper case-based courses. Simulation-based education is recommended for the training of healthcare professionals [4–6]. When compared with high-fidelity simulation, serious games also possess an interesting immersive capacity and may be used to train a large number of healthcare professionals in a limited amount of time with reduced educational resources.

Serious games are video games developed specifically with an educational purpose [7, 8]. They can be computer-based or use more immersive technologies such as virtual reality combined with head-mounted display. Serious games can promote experiential learning, as described by Kolb [9], when they include a 3D realistic environment close to real life. They can be used to train both technical and non-technical skills [10–14]. We developed a serious game, named *LabForGames Warning,* which aims to improve nursing students’ interprofessional communication behavior and their capabilities to detect patient clinical deterioration. Several studies using serious games in healthcare have already been published and have been included in meta-analyses [10–14], but careful analysis shows that serious games are difficult to compare due to the differences in the populations studied, the variety of game designs, topics included, pedagogical objectives and modalities of assessment. Comparative analysis between the educational value of a serious game and a traditional course is already available for cardiac arrest management, trauma triage and other domains, but it is uncertain whether results obtained in one field apply to the others [13–18] . In addition, several studies have used a serious game to train nurses in the detection of patient deterioration, but are characterized by a high level of heterogeneity and sometimes provide neutral results [19–22]. One randomized study, aiming to compare the assessment and the management of clinical deterioration, in which either a virtual patient or a mannequin was used, found that both simulation methods (serious game and high-fidelity) could be effective in improving nursing-student score performance, with no difference between the two methods [23]. More specifically, few studies have assessed how components of clinical reasoning skills necessary to detect patient deterioration are modified by the use of serious games. Most serious games studies use one result of clinical reasoning (treatment, diagnosis or triage) but an objective criterion evaluating all clinical reasoning skills has rarely been used so far [15, 20]. As described by Levett-Jones et al. [24], clinical reasoning is “the process by which nurses collect cues, process the information, come to an understanding of a patient problem or situation, plan and implement interventions, evaluate outcomes, and reflect on and learn from the process”. Furthermore, the positive impact of instructor-standardized debriefing is clearly demonstrated in high fidelity simulation [25] but its place is unclear regarding the use of a serious game [23, 26].

The objective of this study was to compare the respective value of simulation using the above-mentioned serious game with debriefing and a traditional teaching method to improve the clinical reasoning skills necessary to detect patient deterioration in nursing students.

## Methods

### Serious game development

The serious game project was promoted by the simulation center of Paris-Sud University (LabForSIMS) in collaboration with four nursing institutes (Sud Francilien, Perray Vaucluse, Paul Guiraud and Etampes) through a grant from the Ile-de-France Regional Health Agency (ARS). Four virtual clinical cases were created through an iterative dialogue between the pedagogical team and the the software designer (Interaction Healthcare®, Levallois-Perret, France). The pedagogical team were clinical experts (instructors of four nursing schools and anesthesiologists) who were also involved in the simulation center. The predefined educational objectives of the serious game were detection of clinical deterioration and communication. In the game, a nurse was expected to identify clinical deterioration in different clinical situations and warn the medical team appropriately, in consideration of the patient’s clinical severity. As the serious game focuses on nursing students, the objectives had to comply with the French official nursing repository [27]. The game is not played in real time but a clock was presented on the screen which indicated the time flow. In each clinical scenario, three consecutive steps (mildly abnormal, moderate aggravation and severe condition) were constructed to reproduce a specific complication of increasing severity, with the aim of introducing the concept of early warning signs [28]. In the present study, only two of the four available cases were used (Additional file 1):

- Postoperative hemorrhage case: an adult female patient who had undergone a scheduled total hip replacement earlier in the day and who is lying in her ward room bed immediately after arrival from the post-anesthesia care unit. Postoperative hemorrhage is progressively occurring from the surgical site.
- Brain trauma case: an elderly patient with dementia living in a nursing home, whose anticoagulation is associated with progressively developing neurological deterioration after brain trauma from a fall.

Learning safe and standardized communication was an additional educational objective [29, 30]*.* We chose to train nursing students to the SBAR method, (« Situation, Background, Assessment, Recommendation »), adapted into French by the French Health Authority [27].

Before starting to play, a tutorial allowed a first approach to the software. In the game, the player was a nurse. The nurse played on a computer and could move in the 3-D environment (Additional file 1). The student’s actions were obtained by clicking on different interactive areas. For example, regarding clinical examination, the student could check arterial pressure, motor response, etc. Patient questioning was performed by a scroll-down menu of pre-determined options but the player could hear the patient’s answer. The student had also access to the patient’s file (prescription, nurse handover transcripts ...) and could call the physician with a virtual phone and present the case using the SBAR method.

At the end of each scenario, an automatic feedback was presented to the student, which included main guidelines and key messages as well as a global and detailed score according to a grid which had been constructed by the pedagogical team during the development phase of the SG. The student’s actions regarding clinical examination (e.g., checking of arterial pressure, pain assessment...) and the student’ decision (e.g., call for the physician...) were scored using positive, negative or neutral points depending on the different steps of the case. Moreover, positive or negative points were attributed to the quality of the communication during the SBAR tool part of the game. This score and the playtime were not used to evaluate the students in this present study.

### Study description

**Fig. 1:**
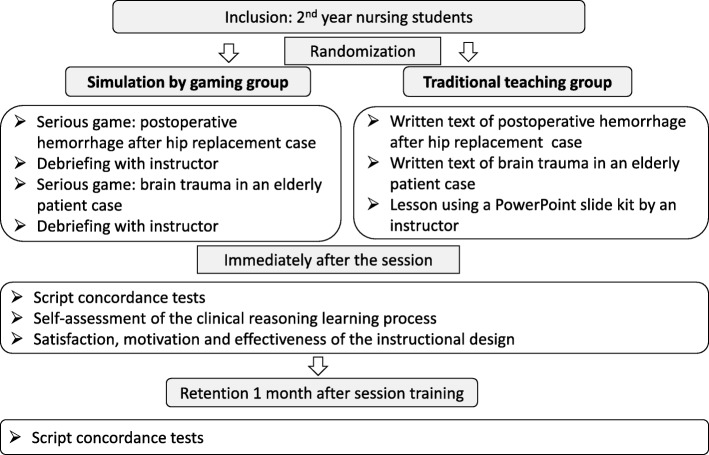
Study design

After informed and written consent, 2nd year nursing students were included and randomized into two groups: the simulation by gaming group (SG group) and the traditional teaching group (TT group) (Fig. 1).

Training sessions for the detection of clinical deterioration were planned in the four nursing institutes. The group sessions lasted 2 h and each involved 15 students. The teachers were nurse instructors involved in the program and trained in simulation. The students had not been informed of the study objectives, nor to which group they were to be assigned (single-blind). Randomization was carried out on the morning preceding the session with the use of the random Excel function (by A.B.). The sessions and assessment for the two groups were run simultaneously in different rooms.

In the SG group, the students played individually on computers and were given the two previously described cases of *LabForGames Warning* (Additional file 1). A whole-group debriefing was performed after the cases by trained instructors. Debriefing (different from serious game’s automatic feedback) was performed according to the methodology used in a simulation session (reaction, analysis and synthesis phases) [25, 31, 32] in which reflexive practice is promoted by a positive interaction between students and instructor.

In the traditional teaching group, the students individually analyzed the same 2 cases which were presented in text paper format (Additional file 2) (without instructor). Then, they attended a PowerPoint slide kit traditional teaching course on the topic with an instructor (Additional file 3).

### Assessment method

All players answered a questionnaire at the end of the session which included questions on sex, age, post-graduate experience and previous video gaming activity (entertainment and professional education). The primary outcome measure was the student’s clinical reasoning skills regarding detection of clinical deterioration as measured by script concordance tests (SCTs) immediately after the session. SCTs have been validated as an effective tool of assessing clinical reasoning skills (Kirkpatrick level 2) and are considered to be an objective and quantitative assessment method. SCTs have already been used in nursing students [33–37]. Based on the script theory [37], SCTs are used to compare if students’ decisions, made from their knowledge networks (scripts), are in line with the decisions taken by a panel of experts. The SCTs used in this study were carefully prepared by following the guideline described by Charlin et al. and Fournier et al. to obtain a high level of fidelity and validity [33–35]. An example of SCT is presented in Table 1. SCTs are case-based tests consisting of short scenarios, and for each the trainee has to interpret newly formulated information against the baseline one to modulate the final decision with the use of a five-point Likert-scale. This final decision could be a diagnosis but also a treatment, an action (prescribe or perform a complementary exam, call for help …). Because the numerous short cases used explore the different types of decision, the SCTs test the different skills of clinical deterioration reasoning and not just the diagnosis.

**Table 1:** Example of script concordance test

N°	If you were thinking of...	And then you find that ...	this hypothesis becomes … (circle your answer)				
1.1	Anticipating the drawing of a blood count	His heart rate is 120 beats/ min	-2	−1	0	1	2
1.2	drawing a capillary blood glucose	His blood pressure is 80 / 40 mmHg	−2	−1	0	1	2
1.3	Calling the physician	He has abdominal pain	−2	−1	0	1	2

You are a nurse in the Surgery Department

A 45-year-old patient who has undergone splenectomy 2 days ago calls you because he feels some discomfort. He has a history of hypertension

− 2 Much less likely

- 1 Less likely

0 Neither more nor less likely

+ 1 More likely

+ 2 Much more likely

The standard SCT scoring method uses the aggregate method in which scoring is in relation to the scores obtained by a panel of reference experts in the respective domain. After scoring the single items, a raw summary score is obtained by summing up all of cases. In order to make the scores interpretable and comparable, the raw summary score is normalized where the expert mean performance is set at 80 points, and the standard deviation at 5 points. This normalization is a Z transformation as suggested by Charlin et al. [38]. The analysis of SCTs includes variance calculation and scores obtained by an aggregation method using the following file (https://www.cpass.umontreal.ca/recherche/groupe-de-recherche-cpass/axes-de-recherches/concordance/tcs/corriger_tcs/). Ninety-three SCTs in connection with the pedagogical objectives were constructed by 3 expert instructors (2 anesthesiologists and 1 nurse instructor). Eleven other experts were subsequently included in the reference panel (6 anesthesiologists and 5 nurse instructors). After analysis of the expert panel responses, 13 SCTs were removed because of high variance (variance > 1) and 80 SCTs were finally used. After optimization of the tool, Cronbach’s alpha was 0.75 (95 CI: 0.70–0.81). The mean SCT score obtained by experts (*n* = 11) was 83.3 ± 3.6 (95 CI: 82.2–84.4). The students were trained to use the SCT format just before the training session. The same 80 SCTs were applied immediately and 1 month after the session, to capture retention of knowledge, with the use of an online module.

In addition, secondary outcome measures were assessed. A self-assessment of the perceived change of the clinical reasoning process was recorded after the session to assess the various steps of nursing clinical reasoning as defined by Levett-Jones et al. [24]. The questionnaire initially described by Koivisto et al. was modified by adding 2 specific questions on clinical deterioration described by Liou et al. [20, 39]. The modified questionnaire was translated into French by the research group (A.B. and D.B.). Each question assesses a specific step of the clinical reasoning process (“I learned to ….” ) using a five-point Likert scale. A global score (graded out of 75) was obtained by adding values given to the 15 questions [20, 24, 39]. Moreover, the students’ perceived satisfaction, their motivation toward learning the specific topic and the effectiveness of the instructional design were assessed by a questionnaire at the end of the session with a Likert scale (1 to 10) corresponding to level 1 and 3 of the Kirkpatrick training evaluation model [40].

### Statistical analysis

The primary outcome was the students’ clinical reasoning skills regarding the detection of clinical deterioration as measured by the SCTs. We assumed that the traditional teaching group would reach an average score of 65/100 together with a standard deviation of 10/100 and, that the new training modality would improve the score by at least one standard deviation (mean difference 10/100 before-after). Accordingly, the sample size was set to 50 students per group using an alpha risk of 5% and beta risk of 10% with a two-sided two sample t-test (R software, https://marne.u707.jussieu.fr/biostatgv/). In view of the risk of attrition, each group was composed of 73 students.

The results are presented as mean ± standard deviation or percentage and confidence intervals. After assessing normal distribution, statistical analysis was performed with the use of parametric tests (Student’s *t* test or Chi^2^ test) (JMP software, SAS institute®). A *p* value less than 0.05 was considered significant. For multiple comparisons, a Bonferroni correction was applied and the statistical significance threshold was lowered to 0.003 (alpha/15).

### Ethical statement

This study was approved by The Institutional Review Board of SFAR (IRB-00010254 − 2017-044). The project had been registered on ClinicalTrials.gov (NCT03428269) [41]. The study was carried out with the use of the CONSORT tool adapted for simulation studies and the GREET Tool for educational studies [42].

## Results

### Participants

**Fig. 2:**
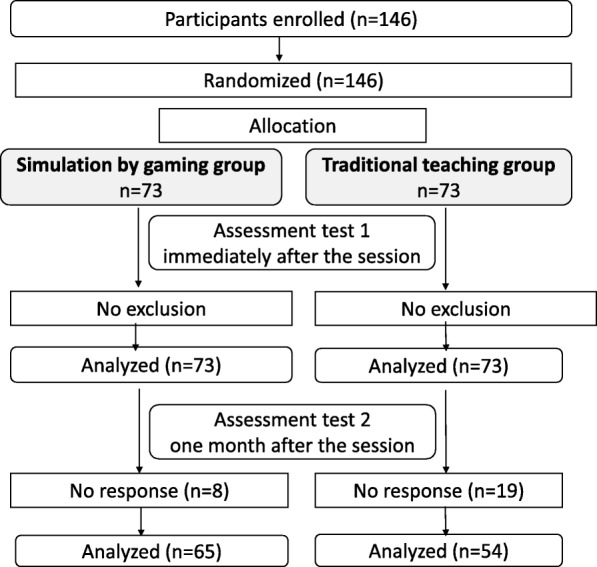
Study flow

Five training sessions were organized in the 4 nursing institutes in February 2018. In total, 146 voluntary nursing students were included and randomized: *n* = 73 in the SG group and *n* = 73 in the TT group. No exclusion was observed (Fig. 2). Participant characteristics are presented in Table 2 and no significant differences were observed. The students of both groups did not differ in their experience regarding the clinical situations presented.

**Table 2:** Participant characteristics

	SG group (*n* = 73)	TT group (n = 73)	*p*
Age (years) (mean ± SD)	24 ± 6.4	25 ± 6.5	0.39
Sex (F/M) n (%)	59/14 (81/19%)	65/8 (89/11%)	0.17
Video gaming activity: *n* (%)			
Never	39 (53%)	43 (59%)	0.15
1/month	11 (15%)	12 (16%)	
1/week	10 (14%)	13 (18%)	
Everyday	13 (18%)	4 (6%)	
No response	0 (0%)	1 (1%)	
Video gaming activity in healthcare: % of players			
Yes	13 (18%)	13 (18%)	0.97
No	59 (81%)	60 (82%)	
No response	1 (1%)	0 (0%)	
Previous experience of clinical situations presented in the course: 1 (no) to 10 (expert)			
Brain trauma	2.7 ± 2.1	2.3 ± 1.8	0.21
Postoperative hemorrhage	3.0 ± 2.4	2.6 ± 2.0	0.39

Results are presented as mean ± standard deviation, or percentage. Comparisons were carried out with the use of the Student’s *t* test on means, or a Chi^2^ test on percentages

*: *p* value less than 0.05 was considered significant

### SCTs results immediately after the session (primary outcome) and 1 month later

**Fig. 3:**
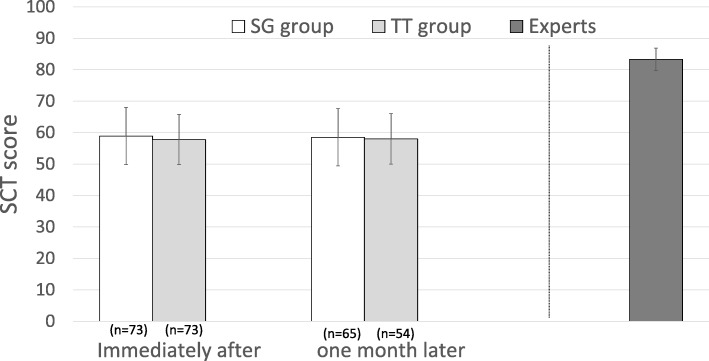
SCT score immediately and 1 month after the session in SG group and TT group

Immediately after the session, the mean SCT scores were 58.9 ± 9.1 in the SG group and 57.8 ± 8 in the TT group with no significant difference (*p* = 0.43) (Fig. 3). One month later, 119 nurse students answered the SCTs (*n* = 119/146): *n* = 65 in the SG group and *n* = 54 in the TT group. The mean SCT scores were 58.5 ± 10.2 in the SG group and 58 ± 9.1 in the TT group (*p* = 0.77) (Fig. 3). Scores obtained immediately after the session and 1 month later were not significantly different between groups.

### Self-assessment of clinical reasoning

Following the training session, all students said that their knowledge of the different steps of the clinical reasoning process had increased. The scores were all above 3.4/5 with no significant difference between groups (Table 3).

**Table 3:** Results of self-assessment of learning the clinical reasoning process between groups

I learned: 1 (not at all) to 5 (very much)	SG group (*n* = 73)	TT group (*n* = 73)	*p*
To collect information			
Collect information by interviewing the patient	4.0 ± 1.0	3.6 ± 1.2	0.02
Collect information by observing the patient	4.2 ± 0.8	4.0 ± 1.0	0.19
Collect information from measurable patient data	4.2 ± 0.8	4.2 ± 0.9	0.76
To process information			
Analyze data to reach an understanding of signs or symptoms	4.3 ± 0.8	4.3 ± 0.8	0.86
To identify problems/issues			
Make nursing diagnosis	4.2 ± 0.8	4.0 ± 0.9	0.10
Recognize possible early signs or symptoms when a patient’s health deteriorates	4.1 ± 0.8	4.1 ± 0.9	0.91
Make decisions on patient care independently	4.1 ± 0.8	3.7 ± 0.8	0.004
Make decisions on patient care in cooperation with other students	3.5 ± 1.2	3.0 ± 1.1	0.75
Make prompt decisions on patient care	3.9 ± 0.9	3.8 ± 0.9	0.30
To establish goals			
Prioritize patient’s need for care	3.7 ± 1.0	3.9 ± 1.1	0.24
Set goals	3.4 ± 1.0	3.4 ± 1.2	0.82
Plan nursing interventions	3.5 ± 1.1	3.6 ± 1.1	0.54
To take action			
Implement nursing interventions	4.1 ± 0.9	4.1 ± 0.7	0.61
Communicate vital information clearly based on the patient’s current condition	4.4 ± 0.8	4.3 ± 0.9	0.42
To evaluate outcome			
Evaluate effectiveness of interventions	3.9 ± 0.9	3.8 ± 1.1	0.44
Total scoring (/75)	59.5 ± 0.9	57.8 ± 1.0	0.25

Results are presented as mean ± standard deviation and comparisons were carried out with the use of the Student’s *t* test. The Bonferroni criterion was set at alpha/15 = 0.003 to reach statistical significance

### Satisfaction, motivation and professional transfer

Students in the SG group significantly expressed significantly more satisfaction toward the training session than those in the TT group (*p* = 0.001) (Table 4).

**Table 4:** Results of satisfaction, motivation and professional impact self-assessment

	SG group (*n* = 73)	TT group (*n* = 73)	*p*
Are you globally satisfied with this training course? 1 (not satisfied) to 10 (very satisfied)	8.5 ± 1.6	7.6 ± 1.7	0.001*
Are you globally satisfied with the educational tool used for case-based learning? 1 (not satisfied) to 10 (very satisfied)	8.5 ± 1.4	8.0 ± 1.6	0.04
Do you think that this training course motivates you to learn? 1 (absolutely not) to 10 (agree absolutely)	8.7 ± 1.9	7.7 ± 2.0	0.003*
Do you think that this training will have an impact on your future professional work? 1 (absolutely not) to 10 (agree absolutely)	8.3 ± 1.8	7.7 ± 1.7	0.06
Would you recommend this training to students or colleagues? 1 (absolutely not) to 10 (agree absolutely)	8.8 ± 1.7	7.8 ± 2.0	0.002*

Results are presented as mean ± standard deviation or percentage. Comparisons were carried out with the use of the Student’s *t* test or a Chi^2^ test. The Bonferroni criterion was set at alpha/5 = 0.01 to reach statistical significance; *: *p *value less than 0.01 was considered significant

Moreover, regarding the pedagogical tool used for training, the students of the SG group expressed more satisfaction than those in the TT group (*p* = 0.04). Students of the SG group perceived the training session as more engaging, as reflected by their significantly increased motivation (*p* = 0.003).

The global educational value (*Would you recommend this training to students or colleagues?)* was more positively significant in the SG group (*p* = 0.002). Both groups declared that the session could have an impact on their future professional work but the difference failed to reach significance (Table 4).

## Discussion

In this study in which we assessed clinical reasoning skills to detect patient deterioration, we found no significant difference between a serious game-based simulation format and a traditional teaching method immediately and 1 month after training. However, the students expressed more satisfaction and motivation with the innovative teaching method.

The education of nurses, who are frontline healthcare providers, is essential to improve the detection of patient deterioration. Serious games dedicated to the same learning objective have already been created by others [19–22]. In Australia, a web-based e-simulation program suggested an improvement of clinical knowledge of patient deterioration and enhanced students’ self-assessed of knowledge, skills, confidence, and competence [19]. Similar results were obtained by Liaw et al. in nurses working on surgical wards [21, 22] and another team also used a serious game to explore the learning process of nursing students ‘clinical reasoning, using a self-assessment questionnaire [20]. However, none of the above-mentioned studies included a traditional educational modality as a control group. Moreover, an objective and quantitative assessment method such as SCT has rarely been used [43].

In education, the learning process can be explored using the learning levels described by Kirkpatrick [40]. Our study showed no difference between the two methods (simulation with serious game vs traditional teaching) with regard to the improvement of clinical reasoning skills necessary to detect patient deterioration (Kirkpatrick level 2). This result was obtained by comparing not only SCT scores, but also by some questions measuring the self-assessment of the different steps of the clinical reasoning process. Our results are consistent with those of Dankbarr et al. and show that serious games and e-modules are equally effective in developing knowledge in patient safety [16]. Drummond et al. also found that training medical students in the management of cardiopulmonary resuscitation with a serious game was as effective as using an online course [17]. Likewise, in a randomized study, Liaw et al. failed to show any difference between virtual patient and mannequin-based simulation in a refresher training course on managing deterioration [23]. By contrast, the randomized study of Mohan et al. evidenced an improved decision-making capacity in trauma triage with the use of an educational video game as compared to app-based training [15]. Other studies have compared the efficacy of different educational methods but in other healthcare domains (such as trauma, urology, surgery, pharmacy) and have also provided conflicting results for learning [15, 18, 44, 45]. In a recent systematic review, serious games used in health professions seem at least effective as a learning control method and may be more effective in improving knowledge and skills [13]. One major limitation of this review was the high level of heterogeneity. The existing studies were difficult to compare and generalizability difficult to reach due to the differences in the populations studied, the variety of the serious game assessment methods, the game designs, and the pedagogical objectives [10–14]. The definition itself of a serious game is sometimes ambiguous between e-learning, virtual patient and serious game [46]. In our study, serious games are defined as video games developed specifically with an educational purpose [7]. Finally, in the 30 randomized clinical trials included in this systematic review, no study assessing the detection of clinical deterioration was included [13]. This is therefore important to better specify the place of the serious games in professional healthcare education and their educative impact, especially on professional practice.

In the studies described above, the clinical reasoning skills were poorly assessed. In most studies, the knowledge or the end-result of the clinical reasoning process (diagnostic or treatment), but not the clinical reasoning process itself, were assessed. In our study, we tried to explore the clinical reasoning skills and all different steps of the clinical reasoning process using firstly a method, i.e. SCT, which has been validated as an effective tool of assessing clinical reasoning skills [33–36, 43]. The SCTs used in our study were related to the content and the educational objectives (clinical deterioration) of the serious games used by students. This was a concern in order to link the strategy to a theoretical learning model of clinical reasoning, the script theory. The final decisions tested with our SCTs explored many different nurse decisions (diagnosis but also treatment or action to perform). SCT is an objective and quantitative assessment method which reduces interpretation bias. Secondly, self-assessment of the different steps of clinical reasoning was obtained with the use of a modified tool [20, 39]. This tool explores the process by which nurses collect information, process the information, identify problems/issues, establish goals, take action, and evaluate outcomes. Recently, and after the beginning of our study, Liaw et al. published a study describing another specific tool used to assess clinical reasoning in clinical deterioration which, on the whole, used the same criteria as our own study [47]. The weaknesses in the previous literature as well as the neutral results (including ours) could be explained by the difficulty to assess clinical reasoning, the complexity of education, and the need to explore results in the long-term.

Learning retention is essential but has been less studied. Similar to our results, Drummond et al. found no difference in the management of cardiopulmonary resuscitation (by means of a mastery learning checklist) 4 months after training of medical students with a SG or an online course [17]. By contrast, the previously mentioned study of Mohan et al. showed an improved decision-making capacity in trauma triage 6 months after initial training with the use of an educational video game compared to traditional apps [15].

As described by Kirkpatrick, satisfaction is the first step of the learning process [40]. In the present study, the students were globally satisfied with the training received and the educational tool used. However, satisfaction was greater in the SG group. Satisfaction associated with the use of a serious game is often described but not always by using a blind comparison design [48, 49]. Boeker et al. reported significantly higher satisfaction in an urology adventure game group compared to a group using a written script [45]. Serious gaming may improve satisfaction compared to traditional learning and other modalities of digital education [13]. Moreover, our students perceived this training session as more engaging and providing significantly higher motivation in the SG group. These results collectively confirm that learners are very motivated to use serious games as they are more engaging, interactive and provide more continuous feedback than traditional learning methods [16, 45, 50, 51] or e-modules [16]. Although motivation and satisfaction are complex psychological processes, the motivational effect is important in education and might be associated with better learning outcomes [8, 52, 53]. Increased satisfaction and motivation when a new pedagogical tool is used could engage the student to learn more, and this could have long-term impact. Active learning is known to increase students’ performance in various scientific domains [54] and this could also be true for simulation by gaming in the medical field.

Professional attitudes have been less studied, and there is limited evidence of a beneficial effect of using a serious game [13]. In the present study, both the SG and the TT group similarly declared that the session could have an impact on their future professional work but no significant difference was found between the two groups (level 3 of the Kirkpatrick training evaluation model).

The present study was original as it included instructor-standardized debriefing after the serious game session in a way similar to debriefing carried out in high fidelity simulation [25]. Our objective was to evaluate a simulation method using serious games and not just the game itself. The importance of instructor-led debriefing is well demonstrated in simulation in medical education [31, 32]. However, paradoxically, its place is extremely limited in the use of serious games. This could be explained by the frequent online use of these games, which is more compatible with digital feedback [16, 19, 26]. In many studies, games are played in the presence of instructors who are able to interact with the players but surprisingly no standardized debriefing is performed [20, 43]. It is therefore unknown if the addition of debriefing to the SG session is of any educational value or not. In our study, digital feedback was included in the serious game, but an instructor-standardized debriefing with the players (reaction, analysis and synthesis phases) was added. Importantly, this did not improve the learning efficacy as results were similar to those obtained after the traditional training method. Additional studies are necessary to seek the efficacy of serious game including or not a debriefing session on clinical reasoning skills .

### Limitations

One limitation in studies such as ours is the difficulty in assessing clinical reasoning skills as it is a complex process. We used SCTs as, in education literature, they are reported to be a well-validated evaluation method [33–36]. Our SCTs were carefully prepared and followed the guidelines described by Charlin et al. and Fournier et al. to obtain adequate fidelity and validity [33–36]. Although our students were trained to use SCTs before the beginning of the session, the training lasted 5 min only and it may be that some students had not fully understood how to correctly answer the questions. Nevertheless, the mean student SCT score (57.8 to 58.9) and the mean expert SCT score (83.3) in our study are similar to those previously described in the literature, which suggests that our use of SCTs was correct [43]. In addition, self-assessment was equally used to assess the various steps of the nursing clinical reasoning process but this part is less validated, as it rests on student’s perception only [20, 24, 39, 47].

An additional limitation may be related to the use of only 2 cases which might have reduced the impact of the training course. However, the SCTs did explore the same theme of clinical deterioration detection in both groups. Thus, the lack of difference between the two groups could be explained by the short duration of training (total: 2 h). In addition, no pretest knowledge was assessed as we expected a learning bias related to the use of the same SCTs. Moreover, the session could not be prolonged for practical reasons, and adding 80 SCTs would have reduced the time dedicated to the training itself. The pretest was not essential to compare the groups before, as both groups of students had the same low level of clinical experience and had the same basic knowledge (2nd year and same curriculum).

## Conclusion

In this study, clinical reasoning was assessed in nursing students immediately and 1 month after a training course dedicated to the detection of patient deterioration. No significant educational difference between training with a serious game-based simulation course and a traditional teaching course was found. However, satisfaction and motivation were greater when the training involved a serious game-based simulation. Simulation by gaming could positively impact the long-term results of nurses’ education on clinical reasoning.

## Additional files

**Additional file 1 **Screenshot of *LabForGames Warning* serious game.

**Additional file 2.** Text paper format of the 2 cases (traditional teaching group).

**Additional file 3.** PowerPoint slide kit of traditional teaching course (traditional teaching group).

## Data Availability

The datasets used and/or analyzed during the current study are available from the corresponding author on reasonable request.

## Abbreviations

CR: Clinical reasoning
SBAR: Situation, background, assessment, recommendation
SCT: Script concordance tests
SG: Simulation by gaming
TT: Traditional teaching
