# Supplementary material for: Comparative value of a simulation by gaming and a traditional teaching method to improve clinical reasoning skills necessary to detect patient deterioration: a randomized study in nursing students
Source: BMC Med Educ. 2020 Feb 19;20:53. doi: 10.1186/s12909-020-1939-6 (PMC7031947; doi:10.1186/s12909-020-1939-6)
Supplement: Supplementary file 2 — Additional file 2. Text paper format of the 2 cases (traditional teaching group). [file 12909_2020_1939_MOESM2_ESM.docx]

**Appendix 2 :** Traditional teaching**:** Text paper format of postoperative hemorrhage case

**Cas clinique 1 : cas orthopédie**

Nous sommes le 10 juin.

Il est 15 h, vous êtes Pauline Leconte, IDE dans le service de chirurgie orthopédique.

Vous accueillez en postopératoire Mme Catherine Toutet, 65 ans, qui a été opérée ce matin d’une prothèse totale de hanche (PTH) droite programmée.

Dans son dossier de soins, vous notez :

Antécédents: AC/FA paroxystique (en rythme sinusal sur l’ECG préopératoire), HTA

Allergies: aucune

Traitement habituel: Amiodarone 200mg/j; Nicardipine LP 50mg/J, Atorvastatine 10mg/j,  Fluindione 1cp/j (arrêt depuis 5 jours sans relais anticoagulant)

Les constantes avant l’intervention étaient :

-PA: 140/60mmHg

-FC: 75 bat/ min

- FR: 12/min,

- Sp02: 100%

- T°:37°

- EVA: 0

Le bilan pré-opératoire retrouvait INR = 1, Hb= 12 g/dl

*1- Vous entrez dans la chambre de la patiente, que lui demandez-vous à l’interrogatoire ?*

*2- Que recherchez vous ? (cochez la ou les réponses exactes)*

- Identitovigilance
- Contrôle de la voie veineuse périphérique
- Surveillance des redons
- Surveillance du pansement
- Surveillance de la motricité
- Surveillance de la conscience
- Surveillance du score de glasgow
- Examen des pupilles
- Surveillance de la diurèse
- Prise des constantes
- Fréquence respiratoire
- Prise de la température

Vous trouvez :

- PA  :110/60 mmHg

-FC : 88 batt/min

-FR  : 12 mvt/min

-Sp02 : 98 %

-T° : 37° C

- EVA 2/10

- Le Redon 1 fixé en déclive: 200 ml (aspiratif) et Redon 2 fixé en déclive: 300 ml (aspiratif)

-Pansement de la cicatrice propre et occlusif

La prescription est la suivante :

*3- Qu’administrez vous ? (cochez la ou les réponses exactes)*

- Paracétamol 1g voie orale
- Nefopam 40mg par voie orale
- Morphine orale 10mg par voie orale
- Amiodarone 200mg par voie orale
- Atorvastatine 20mg par voie orale
- Nicardipine LP 50mg par voie orale
- Enoxaparine 4000 UI/24h en sous cutanée
- Aucun médicament à administrer

*4- Vous avez terminé avec la patiente, que faites vous ? (cochez la ou les réponses exactes)*

- Vous revenez dans la chambre dans 4 heures
- Vous revenez dans la chambre dans 6 heures
- Vous revenez dans la chambre dans 2 heures
- Vous appelez le médecin
- Vous demandez conseille à un collègue infirmier

Vous sortez de la chambre voir un autre patient.

Puis, il est 19 h, vous réalisez le tour de surveillance.

Vous entrez dans la chambre de la patiente.

*5- Que lui demandez-vous à l’interrogatoire ?*

*6- Que recherchez vous ? (cochez la ou les réponses exactes)*

- Identitovigilance
- Contrôle de la voie veineuse périphérique
- Surveillance des redons
- Surveillance du pansement
- Surveillance de la motricité
- Surveillance de la conscience
- Surveillance du score de glasgow
- Examen des pupilles
- Surveillance de la diurèse
- Prise des constantes
- Fréquence respiratoire
- Prise de la température

Vous trouvez :

-PA=105/50 mmHg

-FC= 105 batt/min

-FR =15 mvt/min

-Sp02 99%

-T°=36°5

- EVA 4/10

-pansement avec tâche de sang délimitée

-Redon 1 fixé en déclive : 300 ml (aspiratif

-Redon 2 fixé en déclive: 450 ml (aspiratif) et changé

La prescription est la suivante :

*7- Qu’administrez vous ? (cochez la ou les réponses exactes)*

- Paracétamol 1g voie orale
- Nefopam 40mg par voie orale
- Morphine orale 10mg par voie orale
- Amiodarone 200mg par voie orale
- Atorvastatine 20mg par voie orale
- Nicardipine LP 50mg par voie orale
- Enoxaparine 4000 UI/24h en sous cutanée
- Aucun médicament à administrer

*8- Vous avez terminé avec la patiente, que faites vous ? (cochez la ou les réponses exactes)*

- Vous revenez dans la chambre dans 4 heures
- Vous revenez dans la chambre dans 6 heures
- Vous revenez dans la chambre dans 2 heures
- Vous appelez le médecin
- Vous demandez conseille à un collègue infirmier

*9- Vous souhaitez appeler le médecin, écrivez ce que vous allez lui dire au téléphone :*

Après avoir eu le médecin au téléphone, il vous dit de revenir surveiller dans une heure.

Il est  20 h, vous revenez surveillez la patiente.

*10- Elle n’est pas souriante, que lui demandez-vous à l’interrogatoire ?*

*11- Que recherchez vous ? (cochez la ou les réponses exactes)*

- Identitovigilance
- Contrôle de la voie veineuse périphérique
- Surveillance des redons
- Surveillance du pansement
- Surveillance de la motricité
- Surveillance de la conscience
- Surveillance du score de glasgow
- Examen des pupilles
- Surveillance de la diurèse
- Prise des constantes
- Fréquence respiratoire
- Prise de la température

Vous trouvez :

- PA :85/40 mmHg
- FC:120 batt/min
- FR : 20mvt/min
- T=36,5°
- EVA 4/10

-la surface de la tâche de sang du pansement a dépassée le repère

-Redon 1 fixé en déclive : 400 ml (aspiratif)

-Redon 2 fixé en déclive : 850 (aspiratif)

La prescription est la suivante :

*12- Qu’administrez vous ? (cochez la ou les réponses exactes)*

- Paracétamol 1g voie orale
- Nefopam 40mg par voie orale
- Morphine orale 10mg par voie orale
- Amiodarone 200mg par voie orale
- Atorvastatine 20mg par voie orale
- Nicardipine LP 50mg par voie orale
- Enoxaparine 4000 UI/24h en sous cutanée
- Aucun médicament à administrer

*13- Vous avez terminé avec la patiente, que faites vous ? (cochez la ou les réponses exactes)*

- Vous revenez dans la chambre dans 4 heures
- Vous revenez dans la chambre dans 2 heures
- Vous revenez dans la chambre dans 1 heures
- Vous appelez le médecin immédiatement
- Vous appelez le médecin à la fin de votre tour des patients
- Vous demandez conseille à un collègue infirmier

*14- Vous souhaitez appeler le médecin, écrivez ce que vous allez lui dire au téléphone:*
