# Supplementary material for: Comparative value of a simulation by gaming and a traditional teaching method to improve clinical reasoning skills necessary to detect patient deterioration: a randomized study in nursing students
Source: BMC Med Educ. 2020 Feb 19;20:53. doi: 10.1186/s12909-020-1939-6 (PMC7031947; doi:10.1186/s12909-020-1939-6)
Supplement: Supplementary file 3 — Additional file 3. PowerPoint slide kit of traditional teaching course (traditional teaching group). [file 12909_2020_1939_MOESM3_ESM.pdf]

Détection de l'aggravation d'un patient

Février 2018

LabForSIMS
Laboratoire de Formation par la Simulation et l'Image en Médecine et Santé

Cas clinique orthopédie

2 objectifs pédagogiques

- Détection de l'aggravation de l'état clinique du patient : faire preuve de raisonnement clinique.
- Etablir une communication adaptée et structurée en utilisant l'outil SAED

Détection de l'aggravation de l'état clinique du patient : faire preuve de raisonnement clinique.

- L'infirmier (ère) est en première ligne dans les unités de soins
- L'infirmier (ère) doit être en mesure de **détecter la détérioration de l'état clinique d'un patient**, de façon précoce et « efficace » pour « lancer l'alerte ».

Détection de l’aggravation de l’état clinique du patient : faire preuve de raisonnement clinique.

- Paramètres physiologiques (fréquence cardiaque, pression artérielle, fréquence respiratoire, température, conscience, saturation en oxygène)
- La dégradation d’un ou plusieurs des paramètres physiologiques doit conduire à **rapprocher la fréquence des mesures de surveillance et/ou demander un avis médical**.

- Demander un avis médical en étant en mesure de décrire les anomalies des paramètres physiologiques et leur évolution renforce la qualité de la demande et améliore l’écoute du répondant.

- Des scores combinant les résultats des paramètres physiologiques permettent la détection précoce de l’aggravation d’un patient
- Exemple: score NEWS (National Warning Score)

Paramètres physiologiques	3	2	1	0	1	2	3
Fréquence respiratoire	≤8		9-11	12-20		21-24	≥25
Saturation en oxygène	≤91	92-93	94-95	≥96			
Supplémentation en oxygène		oui		non			
Température	≤35,5		35,1-36	36,1-38	38,1-39	≥39,1	
Pression artérielle systolique	≤90	91-100	101-110	111-219			≥220
Fréquence cardiaque	≤40			51-90	91-110	110-130	≥131
Niveau de conscience				conscient			Grognement, douleur, inconscient

Royal College of Physicians 2012

Scores	Réponse clinique	Risque clinique
0	Pas de changement<br>Surveillance toutes les maximum 12h	faible
1 à 4	Surveillance toutes les 2 à 6h<br>Avis IDE référente pour augmenter la surveillance et/ou décision avis médical	
≥1 score rouge<br>5 à 6	Surveillance horaire<br>Avis médical en urgence	moyen
≥ 7	Surveillance continue<br>Avis médical en extrême urgence<br>Transfert en réanimation	élevé

- Des feuilles de surveillances avec des codes couleurs en fonction de la mesure des paramètres physiologiques peuvent aider à la détection d’une dégradation

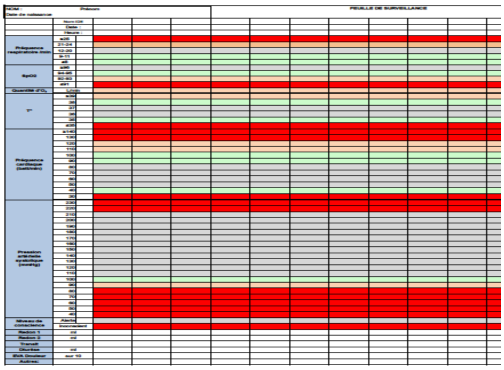

Tachycardie

- Chez l'adulte > 100 batt/min
- Plusieurs étiologies en postopératoire:
  - Signe précoce d'hypoperfusion : hémorragie, sepsis, hypovolémie, insuffisance cardiaque
  - Rétention aigüe d'urine
  - Douleur
  - Fièvre
  - Passage en arythmie cardiaque
  - Embolie pulmonaire
  - Autres : hyperthyroïdie, médicamenteuse (nefopam…).

L'hypotension artérielle

- Pression artérielle systolique < 100mmHg et/ou < 20% de la pression artérielle habituelle
- Etiologies
  - Un choc hémorragique, hypovolémique
  - Un choc septique
  - Un choc d'origine cardiaque
  - Médicamenteuse

- Chez le sujet jeune, en bon état cardiovasculaire, l'aggravation d'une hémorragie aigüe se traduit d'abord par une tachycardie et la baisse de la pression artérielle est souvent tardive.
- **Ne s'inquiéter que lors de l'apparition d'une hypotension peut conduire à un retard diagnostique qui peut avoir des conséquences graves, voire conduire au décès.**
- Dans le cas d'une **hémorragie postopératoire**, l'extériorisation de sang (drainage, pansement) est un signe d'alerte et doit être surveiller

Dans notre cas

- La patiente présente une hémorragie aigüe secondaire à un saignement postopératoire après pose d'une prothèse totale de hanche.
- Initialement, on observe une tachycardie (à 105/min) avec une pression artérielle dans la limite basse (105/50mmHg) avec une augmentation du volume des redons.

= > Cet état nécessite:

- de rapprocher la surveillance,
- rechercher l'apparition d'autres anomalies physiologiques (notamment anxiété ou confusion)
- et de demander d'emblée un avis médical.

Dans notre cas

- Dans un second temps, le volume du saignement continue à augmenter dans les redons et on observe une tachycardie importante (120/min) associée à une hypotension artérielle (85/40 mmHg) traduisant un début d'état de choc hémorragique.

⇒Cet état nécessite:
  - un avis médical en urgence
  - et une surveillance continue.

Etablir une communication adaptée et structurée en utilisant l'outil SAED

- Le **défaut de communication constitue une des principales causes** des événements indésirables en milieu de soins.
- Les différences de personnalité, de culture, de comportement, mais également les **variations intermétiers ou interspécialités** constituent des barrières à la communication entre les professionnels.
- L'utilisation d'outils de standardisation est recommandée pour sécuriser la communication entre les professionnels de santé.

Etablir une communication adaptée et structurée en utilisant l'outil SAED

- L'outil SAED:
  - acronyme signifiant Situation, Antécédents, Evaluation, Demande
  - outil mnémotechnique
  - pour **structurer la communication entre professionnels de santé.**
- L'objectif principal de cette standardisation:

⇒prévenir les événements indésirables pouvant résulter d'erreurs de compréhension lors d'une communication entre professionnels,

⇒mais également de faciliter la mise en œuvre d'une communication documentée claire et concise, et d'éviter les oublis.

Etablir une communication adaptée et structurée en utilisant l'outil SAED

- Cet outil peut être utilisé dans différents types de communication orale :
  - lors de demande orale, d'avis,
  - lors d'une demande urgente,
  - lors d'un transfert
  - ou lors d'un staff.
- Le guide pratique est disponible depuis 2014 sur le site de l'HAS

**S**<br>**Je décris la Situation actuelle du patient**	Assurez-vous préalablement de l'identité de l'interlocuteur appelé.<br>Précisez :<br>– votre identité (nom, prénom, fonction) ;<br>– le service d'où vous appelez ; – la localisation actuelle du patient (unité, service, etc.)<br>– l'identité du patient (nom, prénom, âge)<br>– le motif de votre appel de manière concise ;<br>– la situation actuelle motivant votre appel, basée sur des faits objectifs : signes cliniques, constantes, etc.
**A**<br>**Je décris les Antécédents utiles liés au contexte**	Précisez de manière concise et synthétique :<br>– la date d'admission et le diagnostic posé à l'admission du patient ;<br>– les antécédents médicaux liés à la prise en charge ;<br>– les interventions ou examens invasifs réalisés, liés à la prise en charge ;<br>– les traitements en cours ;<br>– les allergies connues ;<br>– les résultats d'examens pertinents (laboratoire, scanner, etc.) ;<br>– l'état habituel du patient ; depuis quand la situation actuelle a évolué.
**E**<br>**Je donne mon évaluation de l'état actuel du patient**	Donnez votre interprétation de l'évolution de l'état du patient, des constantes vitales, des signes cliniques, et la raison essentielle de votre préoccupation avec des termes précis et clairs. Vous devez penser de manière critique en informant votre interlocuteur de votre évaluation de la situation. Cela signifie que vous devez avoir considéré ce qui pourrait être la cause de l'état de votre patient en vous aidant des différents signes cliniques et résultats d'examens à votre disposition. « Je pense que le problème est … » « Je ne sais pas ce qui se passe mais je suis inquiet »
**D**<br>**Demande**	Exprimez de manière claire, précise et concise votre demande et les délais en termes de temps. « Je souhaiterais …. » « Que dois je faire ? »

- **REPONSE de l'interlocuteur**

➢Il est capital que votre interlocuteur ait compris votre demande d'avis.

➢Une fois que votre interlocuteur a obtenu les informations nécessaires à la bonne compréhension du problème et les réponses à ses questions éventuelles, en réponse à votre demande, il doit :
  - ➢confirmer qu'il a bien compris l'information reçue en la reformulant brièvement
  - ➢puis conclure par sa prise de décision.

➢Ce retour permet de vérifier ensemble que l'information était claire, qu'elle a été bien comprise et que la décision est adaptée.

Dans notre cas clinique: exemple à la fin

S	Je suis Pauline l'infirmière de chirurgie orthopédique. Je vous appelle au sujet de Mme Toutet qui a été opérée ce matin d'une prothèse totale de hanche droite car elle a des redons qui donnent beaucoup, elle est tachycarde et hypotendue. Elle a: FC 120/min, PA 85/40 mmHg et SpO2 96%
A	Mme Toutet a pour antécédent une arythmie cardiaque et une HTA traitées par nicardipine et amiodarone. Tout allait bien en post opératoire immédiat mais depuis quelques heures les redons donnent de plus en plus: 300 et<br>450 mlà 19h et 400 et 850ml actuellement avec un pansement tâché et elle est de plus en plus tachycarde et hypotendue.
E	J'ai l'impression que Mme Toutet est en état de choc hémorragique.
D	Je souhaiterais que vous veniez voir la patiente tout de suite et que vous m'indiquiez ce que je dois faire.

Cas clinique EPHAD

2 objectifs

- Détection de l'aggravation de l'état clinique du patient : faire preuve de raisonnement clinique.
- Etablir une communication adaptée et structurée en utilisant l'outil SAED

Détection de l'aggravation de l'état clinique du patient : faire preuve de raisonnement clinique.

- L'infirmier (ère) est en première ligne dans les unités de soins
- L'infirmier (ère) doit être en mesure de **détecter la détérioration de l'état clinique d'un patient**, de façon précoce et « efficace » pour « lancer l'alerte ».
- Des scores combinant les résultats des paramètres physiologiques permettent la détection précoce de l'aggravation d'un patient
- Des scores plus spécifiques pour évaluer l'état neurologique du patient sont également disponibles.

Détection de l'aggravation de l'état clinique du patient : faire preuve de raisonnement clinique.

- Le **score de Glasgow** est utilisé pour évaluer le **niveau de conscience**
- Il faut rechercher et coter la meilleure réponse motrice, la réponse verbale et l'ouverture des yeux avec un score minimum de 3/15 et maximal de 15/15

**Ouverture des yeux**	
• Spontanée	4
• A la parole	3
• A la douleur	2
• Aucune	1
**Réponse verbale**	
• Orientée	5
• Confuse	4
• Inappropriée	3
• Incompréhensible	2
• Aucune	1
**Meilleure réponse motrice**	
• Obéit aux ordres	6
• Localise la douleur	5
• Retrait à la douleur	4
• Flexion anormale	3
• Extension à la douleur	2
• Aucune	1

Trouble de la conscience ou de confusion

- Les différentes causes à rechercher sont:
  - hypoglycémie: glycémie capillaire ?
  - rétention aigue d'urine: globe vésical ?
  - infection: température ?
  - douleur ?
  - trouble hydro-électrolytique: hyponatrémie ?
  - complication cérébrale (ischémique ou hémorragique): motricité, réponse verbale, chute ?
  - Aggravation d'une démence (diagnostic d'élimination)

Trouble de la conscience ou de confusion

- **L'apparition d'une altération de la conscience:**
  - est une urgence
  - et doit conduire à prévenir un médecin sans délai.

Autres examens neurologiques

- **L'examen des pupilles**

➢ est également à réaliser surtout en cas d'altération de la conscience.

➢ L'apparition d'une mydriase (dilatation de la pupille) aréactive (pas de constriction de la pupille à la lumière) est une urgence neurologique extrême**.**

Autres examens neurologiques

- Pour évaluer **un déficit moteur**:

➢ il faut rechercher une faiblesse musculaire à la mobilisation des 4 membres.

➢ **L'apparition d'un déficit moteur doit conduire à prévenir un médecin en urgence.**

Dans notre cas

- Le soignant constate une dégradation neurologique 48h après un traumatisme crânien chez un patient dément sous anticoagulation curative faisant suspecter une hémorragie intracérébrale.
- Dans le contexte d'un patient dément et âgé, une altération de la conscience modérée (sans déficit moteur) est parfois trompeuse et difficile à diagnostiquer.
- Il faut savoir rapprocher la surveillance et prévenir le médecin en cas de doute.
- Dans notre cas, l'aggravation du trouble de la conscience et l'apparition du déficit moteur doivent conduire à prévenir le médecin en urgence.

Etablir une communication adaptée et structurée en utilisant l'outil SAED

- L'outil SAED:
  - acronyme signifiant Situation, Antécédents, Evaluation, Demande
  - outil mnémotechnique
  - pour **structurer la communication entre professionnels de santé.**

Dans notre cas clinique: exemple

S	Je suis Camille, IDE de l'EHPAD « Les mimosas ». Je suis à la chambre 108. Je vous appelle au sujet de Mr. DONATIEN car il est somnolent.
A	Le patient est en EHPAD depuis 3 ans pour une perte d'autonomie dans le cadre de sa maladie d'Alzheimer. Il a également comme antécédent une valve mécanique sous anticoagulant. Il est noté qu'il a chuté il y a 2 jours sans conséquences. Il est devenu somnolent par rapport à ce matin, il ne répond plus mais n'a pas de déficit moteur. Il a une fréquence cardiaque à 92 et une pression artérielle à 160/90. Il est apyrétique; il présente une plaie mineure du cuir chevelu non suturée.
E	Je pense que ça pourrait être lié à sa chute car il s'est cogné la tête et il est sous anticoagulants. Sinon, c'est peut être dû à une déshydratation aigue, ou à sa démence. En tout cas, Il n'a pas de globe vésical.
D	Pourriez vous venir le voir ? Que dois je faire en attendant ? Voulez-vous que je continue la surveillance ? Est-ce que je dois prévenir sa famille ?

Dans notre cas clinique: exemple au 2ème tour

S	
A	Le patient est en EHPAD depuis 3 ans pour une perte d'autonomie dans le cadre de sa maladie d'Alzheimer. Il a également comme antécédent une valve mécanique sous anticoagulant. Il est noté qu'il a chuté il y a 2 jours sans conséquences. Il est devenu somnolent par rapport à ce matin, il ne répond plus mais n'a pas de déficit moteur. Il a une fréquence cardiaque à 92 et une pression artérielle à 160/90. Il est apyrétique; il présente une plaie mineure du cuir chevelu non suturée.
E	Je pense que ça pourrait être lié à sa chute car il s'est cogné la tête et il est sous anticoagulants. Sinon, c'est peut être dû à une déshydratation aigue, ou à sa démence. En tout cas, Il n'a pas de globe vésical.
D	Pourriez vous venir le voir ? Que dois je faire en attendant ? Voulez-vous que je continue la surveillance ? Est-ce que je dois prévenir sa famille ?

Dans notre cas clinique: exemple au 3ème tour

S	Je suis Camille, IDE de l'EHPAD « Les mimosas ». Je suis à la chambre 108. Je vous appelle au sujet de Mr. DONATIEN car il est somnolent.
A	Comme je vous l'avez signalé il a une maladie d'Alzheimer et une valve mécanique sous anticoagulant. Il est noté qu'il a chuté il y a 2 jours sans conséquences. Quand je vous ai appelé il y a une heure, il était uniquement somnolent, peu réveillable mais maintenant il a un déficit moteur droit est apparu ainsi qu'une mydriase aréactive gauche. Il est hypertendu à 180/90 mmHg avec une fréquence cardiaque à 92/min et il respire normalement avec une Spo2 à 96%.
E	Je pense qu'il a une complication cérébrale peut être liée à sa chute.
D	Pourriez vous venir en urgence? Que dois-je faire en attendant ?
