# Supplementary material for: Comparative value of a simulation by gaming and a traditional teaching method to improve clinical reasoning skills necessary to detect patient deterioration: a randomized study in nursing students
Source: BMC Med Educ. 2020 Feb 19;20:53. doi: 10.1186/s12909-020-1939-6 (PMC7031947; doi:10.1186/s12909-020-1939-6)
Supplement: Supplementary file 1 — Additional file 1 Screenshot of LabForGames Warning serious game. [file 12909_2020_1939_MOESM1_ESM.docx]

**Appendix 1:** Screenshot of *LabForGames Warning* serious game

a. Environment of postoperative hemorrhage case

b. Environment of brain trauma case

c. Player action regarding clinical examination. Example: checking of arterial pressure and postoperative drain.

d. Patient care’s file (prescription, transmission ...)

e. Patient questioning (performed by a scroll-down menu of pre-determined options but the player could hear the patient’s answer)

f. Call the physician (call the physician with a virtual phone and present the case using the SBAR method)

g. Automatic feedback with score
